# Hematopoietic stem cell transplantation in a patient with type 1 mosaic variegated aneuploidy syndrome

**DOI:** 10.1186/s13023-019-1073-x

**Published:** 2019-05-03

**Authors:** Alexandra Laberko, Dmitry Balashov, Elena Deripapa, Olga Soldatkina, Elena Raikina, Alexei Maschan, Galina Novichkova, Anna Shcherbina

**Affiliations:** 1 Dmitriy Rogachev National Center for Pediatric Hematology, Oncology and Immunology, Department of Immunology, 1, Samory Mashela str, 117997 Moscow, Russia; 2 Dmitriy Rogachev National Center for Pediatric Hematology, Oncology and Immunology, Department of Hematopoietic Stem Cell Transplantation, 1, Samory Mashela str, 117997 Moscow, Russia; 3 Dmitriy Rogachev National Center for Pediatric Hematology, Oncology and Immunology, Department of Cytogenetics, 1, Samory Mashela str, 117997 Moscow, Russia; 4 Dmitriy Rogachev National Center for Pediatric Hematology, Oncology and Immunology, Department of Molecular Biology, 1, Samory Mashela str, 117997 Moscow, Russia; 5 Dmitriy Rogachev National Center for Pediatric Hematology, Oncology and Immunology, Medical Department, Moscow 1, Samory Mashela str, 117997 Moscow, Russia

**Keywords:** Mosaic variegated aneuploidy syndrome, *BUB1B* gene., Hematopoietic stem cell transplantation., TCRαβ+/CD19+ graft depletion.

## Abstract

**Background:**

Mosaic variegated aneuploidy (MVA) syndrome is a chromosomal instability disorder that leads to aneuploidies of different chromosomes in various tissues. Type 1 MVA (MVA1) is caused by mutations in the budding uninhibited by benzimidazoles 1 homolog beta *(BUB1B)* gene. The main clinical features of MVA1 syndrome are growth and mental retardation, central nervous system anomalies, microcephaly, and predisposition to cancers. There have been no reports of hematopoietic stem cell transplantation (HSCT) in MVA patients.

**Results:**

We report an 11-year old boy diagnosed with MVA1 syndrome. The *BUB1B* gene mutations c.498_505delAAACTTTA and c.1288 + 5G > A were detected using the next generation sequencing (NGS) method. The patient presented with cytopenia soon after birth, but remained stable until 9 years of age, when he developed myelodysplastic syndrome associated with monosomy of chromosome 7. Due to severe dependence on blood transfusions, a TCRαβ+/CD19+ depleted HSCT was performed from a matched unrelated donor (MUD) using a treosulfan-based reduced intensity conditioning (RIC) regimen. The engraftment occurred, and no severe toxicity was observed soon after the HSCT, but on day + 47, graft rejection was detected. It was followed by prolonged pancytopenia and sepsis with multi-organ *Enterococcus faecium* infection, which led to the patient’s death on day + 156 after HSCT.

**Conclusions:**

In conclusion, we demonstrate that RIC HSCT with TCRαβ+/CD19+ depletion was well tolerated and resulted in complete hematologic recovery in our MVA1 patient, but, unfortunately, it was followed by rapid graft rejection. This fact needs to be taken into consideration for HSCT in other MVA patients.

## Background

Mosaic variegated aneuploidy (MVA) syndrome is a group of rare disorders in which chromosomal instability leads to aneuploidies (predominantly trisomies and monosomies) of different chromosomes in various tissues. Several genetic defects underlying MVA have been described.

MVA type 1 (MVA1) (OMIM #257300) is an autosomal recessive disorder caused by biallelic mutations in the budding uninhibited by benzimidazoles 1 homolog beta (*BUB1B*) gene [1]. Budding uninhibited by benzimidazole-related 1 (BUBR1) protein, which is encoded by *BUB1B* gene, is involved in regulating the mitotic spindle checkpoint during cell division, which maintains the correct number of chromosomes [2, 3]. In mice, full *BUB1B* gene knockout is known to be lethal in embryogenesis [4]. The first MVA1 cases described were caused by compound heterozygous mutations of the *BUB1B* gene [1]. There is also a report of a group of patients with monoallelic *BUB1B* mutations, which are hypothesized to be the cause of their MVA1 because it has been demonstrated that these patients have decreased levels of *BUB1B* transcript and protein [5]. The clinical features of MVA1 syndrome are growth retardation and microcephaly (often detected prenatally using ultrasound [6]), mental retardation, anomalies of the central nervous system (mostly Dandy-Walker malformation), mildly dysmorphic facial features, and predisposition to cancer [1], [5], [7]. The most frequent malignancies associated with MVA syndrome are embryonal rhabdomyosarcoma, Wilms tumor, and acute lymphoid leukemia (ALL), all of which have been reported to manifest in early childhood [ [8, 9]]. The somatic heterozygous mutations of the *BUB1B* gene have also been found to be associated with the development of adult cancers [10–12]. To date, there have been only several reports of MVA1 patients with known genetic defects [1, 5, 6], and to our knowledge, there has been no experience with hematopoietic stem cell transplantation (HSCT) in these patients.

Herein, we present our experience with HSCT in a patient with MVA1 complicated by myelodysplastic syndrome.

## Methods

The two novel mutations of the *BUB1B* gene were found via whole exome sequencing performed using a next generation sequencing (NGS) method. DNA was extracted from EDTA blood using a QIAamp DNA Mini Kit (Qiagen; Hilden, Germany). Libraries were generated according to the manufacturer’s protocols using TruSight One kits (Illumina; San Diego, CA, USA). Sequencing was conducted on the NextSeq500 platform (Illumina). An in-house custom analysis pipeline was used to filter and prioritize variants. The annotations included but were not limited to reference databases from The Genome Aggregation Database (gnomAD), ClinVar pathogenicity annotations, and Online Mendelian Inheritance in Man (OMIM) disorders as gene-based annotations. The mutations were confirmed in the patient using Sanger sequencing, and each mutation was detected in a heterozygous state in the respective parent. The function of the mutant proteins was predicted as being pathological but was found not to have been reported using searches of the Human Gene Mutation Database and the relevant scientific literature.

Mutations of the *NBN* (nibrin) gene had previously been excluded using Sanger sequencing. Karyotyping was performed as previously described [13]. Fluorescent in situ hybridization (FISH) analysis was performed using a Vysis LSI D7S486/CEP7 (Abbott Laboratories; Edmonds, WA, USA) DNA probe according to the manufacturer’s instructions.

The lymphocyte subsets were assessed using standard flow cytometry and monoclonal antibodies (Becton Dickson; Franklin Lakes, NJ, USA) on BD FACSCanto II (Becton Dickson). The levels of serum immunoglobulin were measured using the nephelometry technique on a BN ProSpec (Siemens; Berlin, Germany).

The anti-human leukocyte antigen (anti-HLA) antibodies were assessed using a Luminex multiplex bead array assay and Immucor (Norcross, GA, USA) according to the manufacturer’s instructions. The hematopoietic stem cell graft was derived from peripheral blood mononuclear cell fraction (obtained from matched unrelated donor using apheresis after a standard regimen of stem cell mobilization with granulocyte colony-stimulating factor (G-CSF)). TCRαβ+ and CD19+ graft depletion was performed using a CliniMACS Prodigy instrument (Miltenyi Biotec; Bergish Gladbach, Germany) according to the manufacturer’s recommendations.

For neutrophil and platelet engraftment assessment standard definitions were used [14].

To analyze donor chimerism, whole blood and bone marrow (BM) cells with CD3+ and CD34+ fractions were tested using real-time quantitative polymerase chain reaction assessment of the insertion/deletion short tandem repeats polymorphisms. The graft rejection was confirmed by detecting more than 90% of the patient’s cells in the CD34+ BM fraction.

## Results

We report the case of a boy born in 2005 to healthy nonconsanguineous parents and no relevant family history. At 2 months of age, the boy presented with leukopenia (2.3 х 10*9/l) and thrombocytopenia (80 х 10*/l). Repeated BM investigations during the first year of life revealed BM hypoplasia, two sequential cytogenetic tests demonstrated clones with monosomy 7 with no other clonal anomalies. The patient remained stable and was followed conservatively. At age 9, he developed severe pancytopenia with a granulocytes level < 0.3 х 10*9/л, anemia, and thrombocytopenia and required regular transfusions of red blood cells and platelets. A BM aspirate investigation showed multilineage myelodysplasia with 2–3% of blast cells and 50% of all cells containing monosomy 7. Myelodysplastic syndrome (MDS) which fitted the definition of refractory cytopenia of childhood (RCC) [15] was diagnosed. At age 10, the patient was admitted to our Center. His phenotypic features included microcephaly, growth retardation (height – 2nd percentile, weight – 3rd percentile), facial anomalies (frontal bossing, a triangular face, and micrognathia), multiple areas of skin café au lait hyperpigmentation, alopecia, and mental retardation. Magnetic resonance imaging of the brain detected the Dandy-Walker malformation. Complete blood count and all lymphocyte subsets were proportionally decreased, and dysgammaglobulinemia was demonstrated (Table 1). Because of microcephalia, cytopenia and Slavic origin of the patient, Sanger sequencing on the *NBN* gene (Nijmegen breakage syndrome) was performed, no mutations were found. The patient’s profound cytopenia precluded correct interpretation of the diepoxybutane test due to low number of mitoses.

**Table 1:** Lymphocyte Subsets and Serum Immunoglobulin Levels in MVA1 Patient

	Patient’s levels	Normal ranges
CD3+, х10^a^9/l	0.297	1.4–2,0
CD4+, х10^a^9/l	0.159	0.7–1.1
CD8+, х10^a^9/l	0.05	0.6–0.9
CD19+, х10^a^9/l	0.042	0.3–0.5
CD3-CD16 + CD56+, х10^a^9/l	0.063	0.1–1.3
IgA, g/l	3.62	0.9–1.9
IgM, g/l	2.66	0.8–1.9
IgG, g/l	5.79	8.7–11.7
Hemoglobin^a^, g/l	57	115–140
Platelets^a^, х10^a^,9/l	0	150–400
White blood cells, х10^a^9/l	0.42	6–9.8
Neutrophils, х10^a^9/l	0.02	1.5–8.5
Monocytes, х10^a^9/l	0.04	0.09–0.6

^a^ *minimal level before transfusion*

Although the patient’s underlying genetic defect was unknown at the time, HSCT was considered as the only option to cure the MDS. During his lifetime, the patient received multiple blood transfusions, and positive anti-HLA antibodies were found. Before the HSCT, rituximab (375 mg/m^2^) was given once per week four times. It decreased the frequency of platelet transfusions, but patient remained cytopenic with hypoplastic BM, with blast cells level 0,8-1,6%. The conditioning regimen included treosulfan (30 g/m2), fludarabin (150 mg/m2), and thymoglobulin (5 mg/kg). According to the institutional protocol, the peripheral stem cells from a 10/10 HLA matched unrelated donor (MUD) after TCRαβ+/CD19+ graft depletion procedure were infused. Following depletion the graft contained: nuclear cells 4,33 х10*8/kg, CD34+ cells 9,4 х10*6/kg, CD3+ cells 5,1 х10*6/kg, TCRαβ+ cells 18 х10*3/kg.

Post-transplant immunosuppression consisted of tacrolimus from day − 1 (planned to be administrated till day + 45). After the HSCT, the patient developed mild treosulfan-related toxidermia and had a mild increase in liver enzymes, which resolved after a short pause in the tacrolimus therapy, but no signs of other organ toxicity. The platelet engraftment occurred on day + 16 and the neutrophil engraftment on day + 21 after the HSCT. The maximum platelet and neutrophil counts after engraftment were 129 х10*9/l and 0,72 х10*9/l, respectively. On day + 30 after the HSCT, the patient had mixed BM cell chimerism, with total of 48% of the donor cells, in CD34+ − 4% of the patient’s cells, in CD3+ 88% of the patient’s cells. On day + 47, graft rejection was detected, followed by prolonged pancytopenia with absolute neutrophil count 0, high dependence on blood transfusions (daily platelet and one in 2–3 days red blood cell transfusions), and no response to hematopoietic stimulating factors (G-CSF – filgrastim and thrombopoietin receptor agonist – romiplostim). BM investigation demonstrated aplastic features and no cells containing monosomy 7. Interestingly, after the HSCT, the resolution of the patient’s alopecia was observed.

In parallel, whole exome sequencing was performed using patient’s stored pretransplant blood sample. The results obtained after the HSCT showed two heterozygous mutations in the *BUB1B* gene: c.498_505delAAACTTTA and c.1288 + 5G > A. These mutations have not been described previously, yet the frame shift (c.498_505delAAACTTTA) and splice site (c.1288 + 5G > A) mutations were predicted to be pathogenic. Investigation of the patient’s fibroblasts revealed a normal karyotype and no cytogenetic abnormalities. Mutations in the genes *SAMD9* and *GATA2*, which are frequently associated with monosomy 7 and MDS in early childhood [16, 17] were not detected by exome sequencing of the patient’s pre-HSCT blood sample. Yet *SAMD9L* gene was not tested.

After the graft rejection, a second transplantation from another MUD was planned, but the patient developed sepsis and multiorgan *Enterococcus faecium* infection and had no response to a combination of antimicrobial therapy and donor granulocyte transfusions. Unfortunately, the patient died of infectious complications on day + 156 after the HSCT.

## Discussion

MVA1 is a rare condition caused by *BUB1B* mutations, which were found in our patient in the compound heterozygous state. Although MVA1 patients are highly predisposed to malignancies, to our knowledge, so far, there has been only one report of myelodysplasia in an MVA1 patient [8]. The patient described by Jacquemont et al. had severe cytopenia and signs of myelodysplasia in the BM, which were detected in the first days of life, and multiple trisomies and monosomies in the fibroblasts, BM, and peripheral blood cells. At age three, that patient developed acute lymphoid leukemia and died three months later of the disease’s progression. Interestingly, our patient had remained cytopenic for several years with persistence of the clone with monosomy 7 and no evidence of progression to acute leukemia. Therefore, in our patient, the indications for HSCT were severe BM aplasia and high dependence on transfusions of blood components, rather than long-lasting monosomy 7 associated myelodysplasia.

Despite the fact that his genetic defect was unknown at the time, based on his clinical features and microcephaly our patient was suspected to have had one of the chromosomal instability syndromes. Hence, we performed an HSCT with a reduced intensity conditioning (RIC) regimen and a TCRαβ+/CD19+ graft depletion. Importantly, after the HSCT, no significant toxicity was observed, which would be expected in patients with chromosomal instability syndromes [18]. Unfortunately, on day + 47 after the HSCT, graft rejection occurred. The graft rejection might have been caused by the RIC being not immunoablative enough to clear patient’s residual lymphocytes. The persistence of the anti-HLA antibodies were also considered to be a factor that contributed to the graft rejection. In our previous study of TCRαβ+/CD19+ depleted HSCT in patients with primary immunodeficiencies high incidence of graft rejection after RIC regimens with one alkylating agents was observed [19]. We also have reported 3 of 15 patient with Nijmegen breakage syndrome to reject the graft after Fanconi anemia adopted conditioning regimen [20]. However, this group of patients was too small to evaluate the role of depletion procedure in the graft failure.

Despite the clearance of monosomy 7 clone, after graft rejection the patient failed to recover his hematopoiesis and remained profoundly cytopenic for the next five months. The explanation for this phenomenon could be preexisted long-term BM aplasia with an exhaustion of hematopoiesis, as well as prolonged infectious complications after the HSCT. We also consider the contribution of defective function of the BM precursors, that carried the *BUB1B* mutations. The important role of BUBR1 in hematopoiesis has previously been demonstrated in the *BUB1B* hypomorphic allele (H/H) mouse model [21]. Interestingly, although the patient had been considered as having no obvious clinical signs of immunodeficiency before the HSCT, his alopecia resolved after transplantation, likely proving its autoimmune pathogenesis.

Cytogenetic anomalies were found in our patient’s bone marrow cells, as they were in the hematopoietic and somatic cells of the patients reported previously. Therefore, we conducted a cytogenetic analysis of our patient’s fibroblasts, which revealed no aneuploidy. This fact can be explained by the variegation of the aneuploidy in different cells in MVA1 patients [5].

A variety of primary immunodeficiencies and congenital BM failure syndromes (predominantly with DNA repair impairment), including Nijmegen breakage syndrome, Ligase 4, Cernunnos, Rad50 deficiencies, Fanconi anemia, and some others, combine the symptoms of microcephaly and cytopenia [22, 23]. Most of these syndromes are characterized by high predisposition to malignancies. In these patients, HSCT is a well-established curative treatment for immunodeficiency and/or BM failure, and it also has been shown to reduce the risks of development or relapse of lymphoid or myeloid malignancies [20]. Because the patients with the aforementioned syndromes frequently have increased organ toxicity after myeloablative conditioning due to the abnormal DNA repair, the use of RIC regimens has demonstrated better survival after transplantation [18, 24]. However, the visceral toxicity of a conditioning regimen may be quite different in patients who have phenotypically overlapping, but genetically different, chromosomal-instability syndromes, with some patients tolerating well myeloablative regimens that are nearly full dose [20]. Given this, it is critical to know the diagnosis prior to the HSCT in order to choose a maximally myeloablative and a minimally toxic conditioning regimen, especially in the context of T-cell depletion, which confers a higher risk of rejection in patients with fully or partially preserved immunity. Thus, the next generation sequencing is the method of choice for confirming diagnoses in so variable a group of syndromes, as this case demonstrates.

## Conslusion

In conclusion, we report the first experience with an HSCT in a patient with mosaic variegated aneuploidy 1 syndrome and myelodysplasia who had a good tolerance for the reduced intensity conditioning regimen but who unfortunately experienced graft rejection later.
